# Complex responses of spring vegetation growth to climate in a moisture-limited alpine meadow

**DOI:** 10.1038/srep23356

**Published:** 2016-03-17

**Authors:** Hasbagan Ganjurjav, Qingzhu Gao, Mark W. Schwartz, Wenquan Zhu, Yan Liang, Yue Li, Yunfan Wan, Xujuan Cao, Matthew A. Williamson, Wangzha Jiangcun, Hongbao Guo, Erda Lin

**Affiliations:** 1 Institute of Environment and Sustainable Development in Agriculture, Chinese Academy of Agricultural Sciences, Beijing 100081, Beijing, China; 2 Key Laboratory for Agro-Environment & Climate Change, Ministry of Agriculture, Beijing 100081, Beijing, China; 3 John Muir Institute of the Environment, University of California, Davis, CA 95616, USA; 4 College of Resources Science and Technology, Beijing Normal University, Beijing 100875, China; 5 Nagqu Grassland Station, Nagqu Agriculture and Animal Husbandry Bureau, Nagqu 852100, Tibet Autonomous Region, China

## Abstract

Since 2000, the phenology has advanced in some years and at some locations on the Qinghai-Tibetan Plateau, whereas it has been delayed in others. To understand the variations in spring vegetation growth in response to climate, we conducted both regional and experimental studies on the central Qinghai-Tibetan Plateau. We used the normalized difference vegetation index to identify correlations between climate and phenological greening, and found that greening correlated negatively with winter-spring time precipitation, but not with temperature. We used open top chambers to induce warming in an alpine meadow ecosystem from 2012 to 2014. Our results showed that in the early growing season, plant growth (represented by the net ecosystem CO_2_ exchange, NEE) was lower in the warmed plots than in the control plots. Late-season plant growth increased with warming relative to that under control conditions. These data suggest that the response of plant growth to warming is complex and non-intuitive in this system. Our results are consistent with the hypothesis that moisture limitation increases in early spring as temperature increases. The effects of moisture limitation on plant growth with increasing temperatures will have important ramifications for grazers in this system.

Spring phenology is one of the main indicators of the terrestrial ecosystem response to climate change. Phenological changes are highly dependent on both ecosystem and species^1^. Researchers have used three main approaches to explore the patterns of plant phenological responses to climate change: time series of field observations, correlational studies using remotely sensed data and climate records, and experimental warming studies^2^,^3^. Temporal advances in spring growth have been observed in many systems in the northern hemisphere over the past three decades, particularly in high latitude and alpine regions over the past three decades^4^,^5^,^6^. Such advances in spring vegetation growth can alter the temporal availability of resources such as light, water, and nutrients^7^, but may also alter local climate feedbacks through both physical (e.g., surface albedo) and biological effects (e.g., transpiration, photosynthesis, and carbon storage)^8^.

Studies directed towards the development of a mechanistic understanding of phenology have implicated a variety of critical cues in the control spring phenology, including winter chilling, the photoperiod, and temperature^7^,^9^. Other researchers argue that precipitation and snow cover are also important in regulating spring green-up processes^10^,^11^,^12^. Shen *et al*.^13^ showed that preseason precipitation is an important regulator of the spring phenological response to climatic warming. Spatially, an increase in precipitation of 10 mm corresponds to an increase in the temperature sensitivity of greening of 0.25 day °C^−1^ (ref. 14). Other observational studies have suggested that soil water availability is also important in triggering plant growth in spring^15^,^16^,^17^.

The Qinghai-Tibetan Plateau, known as the ‘roof of the world’, is a 2.5 million km^2^ region dominated by alpine grassland ecosystems^18^,^19^. This alpine grassland is an extremely fragile ecosystem and appears particularly sensitive to climate change^20^. The region contains the headwaters of Asia’s most important rivers, supplying water to almost 1.5 billion people. It also supports the livelihoods of 6.5 million Tibetans who depend on the ecosystem for grazing. This grassland ecosystem also supports to globally endangered grazers, such as the Tibetan antelope and wild yak.

The Qinghai-Tibetan Plateau has experienced a dramatic change in climate in recent years with increasing temperatures^21^,^22^. Ecological researchers have been particularly mindful of the degree to which this warming may affect the date of the alpine grassland within the region. Remotely sensed data for the region, readily available from the National Oceanic and Atmospheric Administration, have been used to observe changes in greenness on the Qinghai-Tibetan Plateau using the Normalized Difference Vegetation Index (NDVI)^13^,^23^,^24^. These studies have shown surprising results. One suite of studies demonstrated an advance in spring phenology on the Qinghai-Tibetan Plateau from early 1980s to the late 2000s^24^,^25^. These researchers considered higher winter and spring temperatures to be the main factors driving the phenological advance of spring. In contrast, other researchers argue that the advancement of the spring green-up date has been reversed since 2000, with a significant delay in the onset of the spring phenological phases^18^,^23^,^26^. Various authors hypothesized that the observed delays in the spring phenology may be driven by: (a) longer times required for chilling, or vernalization, caused by warmer winter temperatures^23^; (b) anthropogenic factors, such as grassland degradation^27^; (c) complex thaw–freeze processes associated with warming^27^; or (d) or drought^28^.

The Qinghai-Tibetan Plateau is a cold environment in which temperature is likely to be a factor limiting plant growth. However, this region is also characterized by dry winters and summer monsoonal precipitation. Therefore, spring vegetation growth may be delayed and constrained by warming until the summer precipitation provides adequate soil moisture to sustain plant growth. An emerging conceptual model of the impact of climate change on this region maintains that warming will advance spring greening and increase plant growth when sufficient water is available, but without adequate springtime precipitation, warming will increase moisture stress and delay spring greening^15^,^29^. We specifically evaluated the hypothesis that temperature and moisture interact to control spring vegetation growth.

To test this hypothesis, we conducted both regional and field studies on the central Qinghai-Tibetan Plateau. We used NDVI to identify correlations between climate and vegetation greening on the regional scale. We also used an open top chamber (OTC) to simulate warming in an alpine meadow on the central Qinghai-Tibetan Plateau. We measured the net ecosystem CO_2_ exchange (NEE) as our response variable, gauging plant growth as carbon uptake. The experiment was established late in 2011 and we collected responses through the 2012, 2013 and 2014 growing seasons. The goal of our study was to better understand the variations in spring vegetation growth in response to climate.

## Results

### Regional Assessments of Change

#### Recent climate change on the central Qinghai-Tibetan Plateau

**Figure 1:**
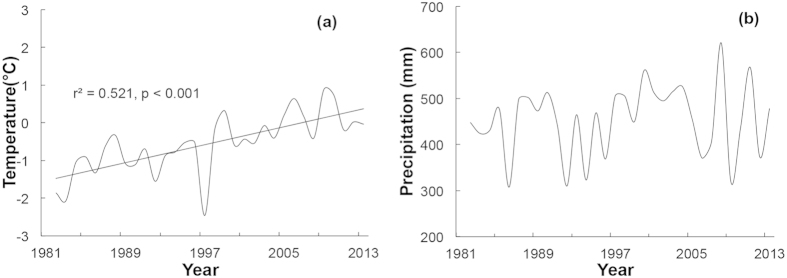
(**a**) Mean annual temperature and (**b**) annual total precipitation from 1982 to 2013 on the central Qinghai-Tibetan Plateau (climate data of Nagqu County).

The climate of the central Qinghai-Tibetan Plateau has been warmed by approximately 2.5 °C of the mean annual temperature since 1982, at an estimated rate of 0.6 °C per decade (Fig. 1, Table 1). However, the mean annual precipitation did not show a significant changing trend from 1982 to 2013 (Fig. 1, Table 1). The aridity index (AI), calculated as the ratio of total precipitation (P) to temperature (T) plus 10 [P/(T + 10)]^30^, also did not change significantly from 1982 to 2013 (Table 1).

#### Regional phenological changes related to climate

**Figure 2:**
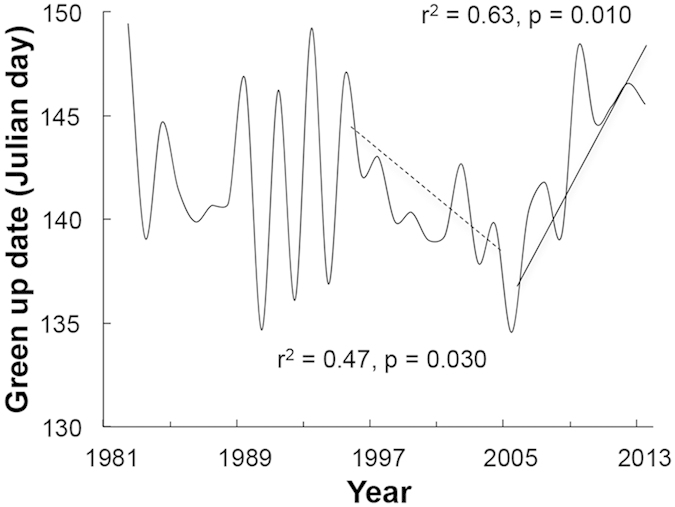
Spring green-up date on the central Qinghai-Tibetan Plateau (Nagqu county) from 1982 to 2013 (Data from AVHRR). There exist three trends of the green-up date: (1) from 1982 to 1994, no change; (2) from 1995 to 2004 (hashed line), advanced significantly (*y* = −0.60*x* + 144.4, *r*^2^ = 0.47, *p* = 0.030); (3) from 2005 to 2013 (solid line), delayed significantly (*y* = 1.26*x* + 136.7, *r*^2^ = 0.63, *p* = 0.010).

**Figure 3:**
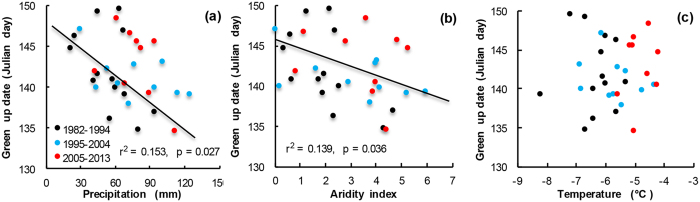
Correlation between winter/springtime (Nov to May) (**a**) total precipitation, (**b**) aridity index in May and (**c**) mean temperature and the green-up date on the central Qinghai-Tibetan Plateau.

The spring greening on the central Qinghai-Tibetan Plateau shows significant inter-annual variations, but has shown no consistent changing trend from 1982 to 2013 (Fig. 2). However, within this time period, the spring green-up date advanced significantly between 1995 and 2004 and was significantly delayed between 2005 and 2013 (Fig. 2). Across this entire time period, the green-up date correlated negatively with the total precipitation in the preseason (winter and spring) and with AI in May, but not with temperature (Fig. 3, Table 2).

### Experimental Warming Impacts on Vegetation Growth

#### Effects of soil temperature and moisture treatments

**Figure 4:**
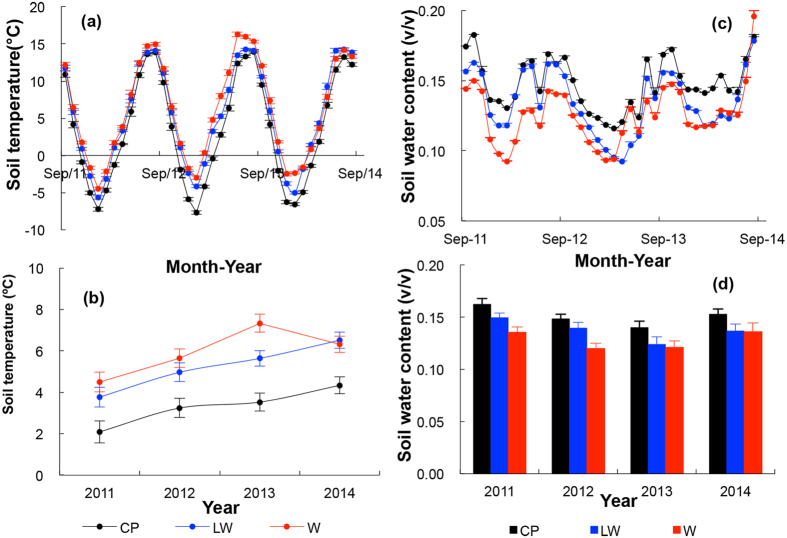
(**a**) Monthly and (**b**) annual soil temperature ( ± SE) and (**c**) monthly and (**d**) annual soil water content ( ± SE) in each treatment from 2011 to 2014. Black symbols represent control plots (CP); blue and red represent limited warming (LW) and warming (W) treatments, respectively.

Throughout the duration of our experiment, from July 2011 to 2014, the soil temperature was significantly higher in the the treatment plots than in the control plots in both the growing (May–September) and non-growing seasons (October–April) (*p* < 0.05, Fig. 4a,b). Across the entire period, the soil temperature increased by 2.0 °C and 2.8 °C, in our limited warming (LW) and warming (W) plots, respectively (*p* < 0.05), showing approximately the same magnitude of change experienced in the region since 1982 (Fig. 1). The temperature differences remained consistent through winter and summer (Fig. 4a).

The soil moisture was lower in the LW and W plots than in the control plots in both the growing and non-growing seasons (*p* < 0.05, Fig. 4c,d). The soil moisture decreased by 8.8% and 15.1% in LW and W plots, respectively, relative to that in the control plots (*p* < 0.05).

#### NEE

**Figure 5:**
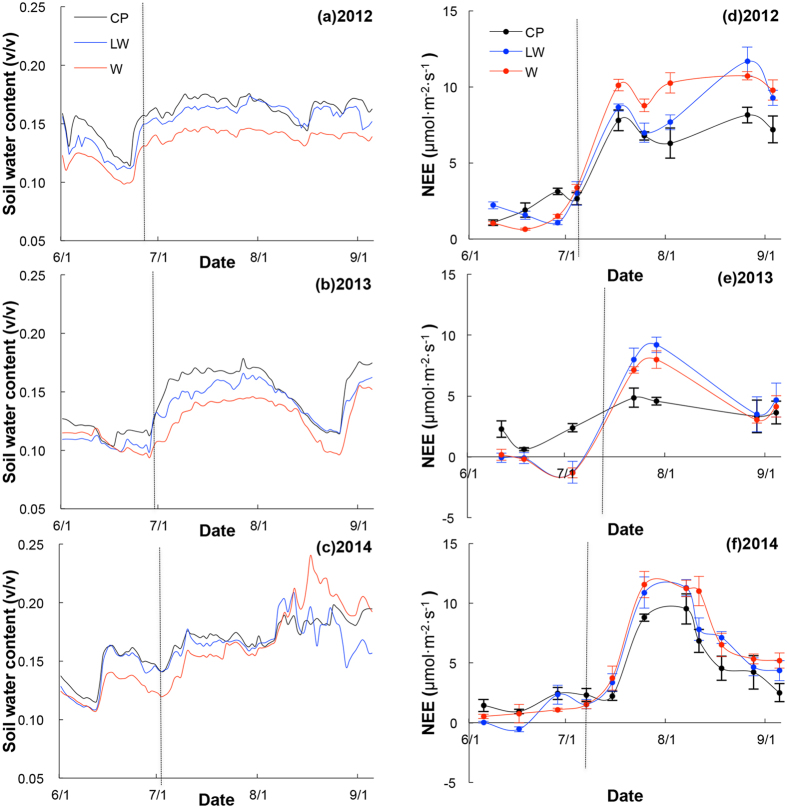
Seasonal variation of (**a–c**) soil water content and (**d–f**) NEE ( ± SE) in treatment and control plots. The hashed line separated plus or minus NEE changes to two periods.

Our results showed that the pattern of soil water content constrained NEE (Fig. 5). We defined the early growing season (EGS) as the period before the initiation of the summer monsoon. In the early growing season, NEE remained low under low soil moisture conditions (Fig. 5). After the monsoon commenced, NEE increased dramatically with the increase in the soil water content.

However, in each year, the soil water content was lower in the treatment plots than in the control plots throughout the growing season (except in August 2014, Fig. 5a–c). Similarly, the onset of consistent positive NEE values varied across years. Consistent with the increased soil water content, these consistent NEE values >0 arrived earliest in 2014 and latest in 2013 (Fig. 5d–f). The model selection results based on random slope–mixed effects models indicated that the models that included aspects of both soil moisture and soil temperature outperformed those models containing only one or the other variable (Table 3). The *Z*-scores for the model-averaged parameter estimates in the early-season model set also indicated that soil moisture (positive effect) and the change in soil moisture between measurements (negative effect) were the strongest predictors of NEE (Table 2).

Once NEE increased to a high value (e.g., >4 μmol m^−2^ s^−1^), it remained higher in the warming plots than in the control plots. This observation confirms our expectations that once water is no longer limiting, temperature becomes limiting, and increased temperatures result in increased growth rates. Similar to the early-season results, the best-performing models of the late growing season (LGS) indicated that a combination of soil moisture and soil temperature (in the form of the interaction between soil moisture and temperature) better predicted NEE than the models including only soil moisture or only soil temperature (Table 3). The *Z*-scores for the model-averaged parameter estimates in the LGS model set indicated that soil moisture was the strongest predictor of NEE (Table 4).

#### Plot level correlations between NEE and soil environmental factors

**Figure 6:**
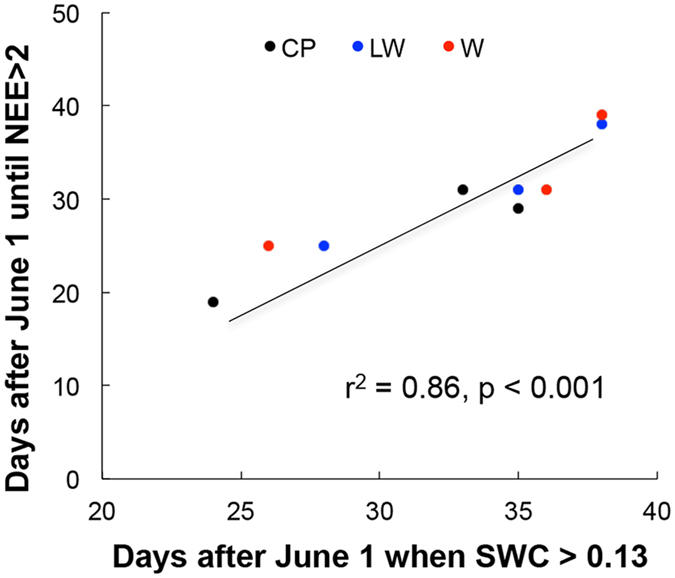
A scatterplot and regression of the average number of days after June 1 that plots attained active spring physiological response (NEE > 2.0 μmol m-2 s-1) as a function of the average date after which soils became moist ( > 0.13 SWC v/v). Symbols represent different experimental plot treatments across the three years of the experiment. Each point represents the average of four replicate plots.

Our experimental plot data demonstrate a clear relationship between the availability of moisture and the onset of springtime. We selected a threshold value for soil water content (>0.13 v/v) representing a transition from a dry winter to a moist spring (Fig. 2) and a threshold value for NEE (>2 μmol m^−2^ s^−1^) that represents the onset of plant growth (Fig. 5). These thresholds were selected visually, but represent the inflection points of sharp increase soil water content and NEE in most years (Figs 2 and 5). The average date upon which this threshold level of physiological activity was reached was strongly predicted (*r*^2^ = 0.86; *p* < 0.001) by the average date of wetted spring soils (Fig. 6).

**Figure 7:**
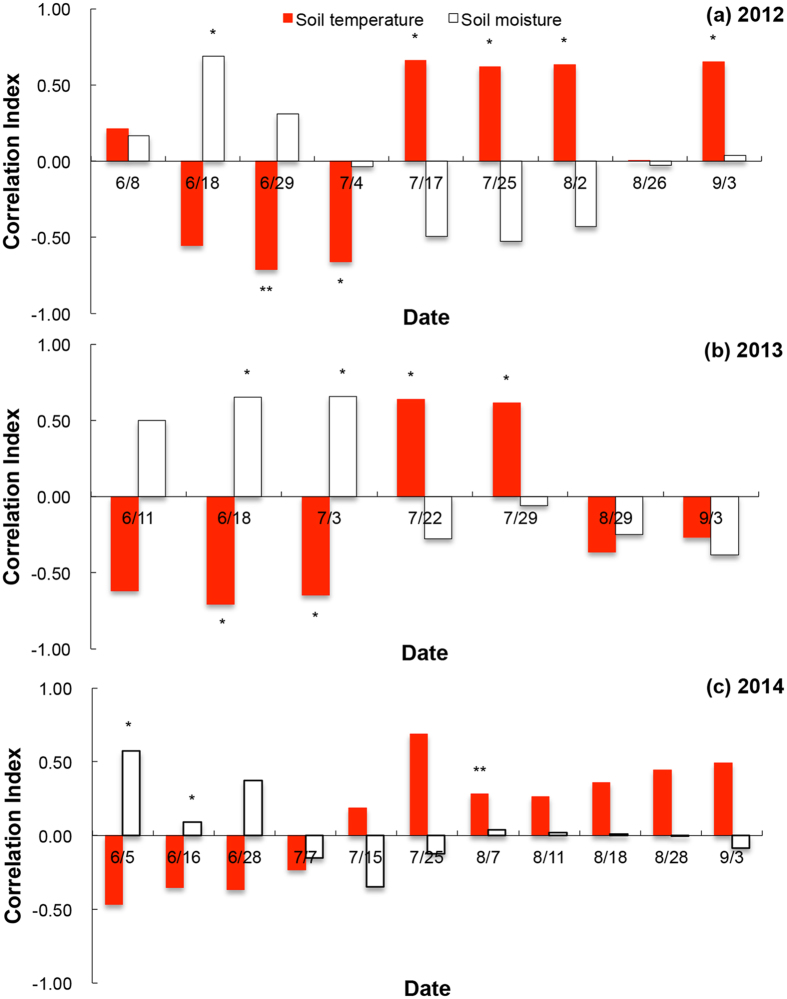
Correlations between NEE and soil temperature and soil moisture in (**a**) 2012, (**b**) 2013 and (**c**) 2014. ‘*’ indicates significantly differences under 0.05 level; ** indicates significantly differences under 0.01 level.

Examining this relationship more closely, we found that the correlations between NEE and soil temperature and moisture varied through the growing season (Fig. 7). During the EGS (left of the hashed line in Fig. 7), NEE correlated primarily negatively with soil temperature and positively with soil moisture, whereas later in the season, the opposite correlations occurred (Fig. 7). In fact, the warmed plots regularly had higher NEE values than the control plots after the ecosystem became wet in the mid-to-late growing season (Fig. 7).

## Discussion

Plant phenological and net growth responses to climatic change are complex and variable^1^. Low temperatures generally constrain plant primary production in cold grassland ecosystems^31^. The Qinghai-Tibetan Plateau, with a below-freezing mean annual temperature, is a cold grassland ecosystem. It is also characterized by a summer monsoon, which delivers over 90% of the annual rainfall during the summer months, making this region a global outlier in the seasonal distribution of moisture among cold grassland environments. We examined the hypothesis that soil moisture limits plant growth because delayed spring greening has been observed throughout the region in recent years.

With warming temperatures, dry winters, and variable precipitation, we anticipated that increasing aridity would delay the onset of spring. The increasing variability also suggested that the spring onset dates may become more variable as well, with some winters retaining enough moisture to overcome the drying effects of increasing temperatures. The inter-annual variability in net rainfall may be quite high, but the strongest driver of the arrival of spring appears to be the timing of the summer monsoon. Our results of warming experiment showed the inter-annual variation in the timing of NEE >0, which differed between 2014 with a wetter spring and 2013 with a dryer spring (Fig. 5). Shen *et al*.^14^ also found that spring greening was more sensitive to inter-annual variations in preseason precipitation in arid areas than in wetter areas.

Our experimental data contribute to the debate by identifying the mechanisms associated with recent observations of delayed spring phenology^18^,^23^. Various researchers have focused on a suite of mechanisms that could drive delayed spring phenology. These include spring cooling^18^, failure of necessary chilling^23^, grassland degradation^27^, and delayed or declining springtime precipitation^13^,^14^. Our experimental work strongly supports the hypothesis that moisture is often limiting. This hypothesis states that moisture often, but not always, constrains the initiation of spring greening when soil moisture is low even though temperature is sufficiently high to stimulate plant growth. Our regional observations of greening, measured with NDVI, showed that precipitation is a much stronger predictor of greening than temperature (Fig. 3).

Supporting the moisture limitation hypothesis, Dorji *et al*.^15^ found that the addition of snow advanced the spring plant growth in alpine meadow on the Qinghai-Tibetan Plateau, whereas warming alone delayed the grassland phenology. Similarly, Wang *et al*.^32^ found that the timing of phenological events advanced with elevation, and that higher temperatures with earlier snowmelt at low elevations delayed spring events on the Qinghai-Tibetan Plateau. On the central Qinghai-Tibetan Plateau, climate change is driving warmer temperatures, with the mean annual temperature now exceeding 0 °C (Fig. 1), so climate change may potentially increase ecosystem productivity. However, the arrival of spring rains strongly constrains this potential increase. Our results showed a strong correlation between soil moisture and plant productivity across experimental treatments and years (Figs 5 and 6). Although the increasing of temperature accelerates plant growth, warming can also cause soil moisture to decline. The combined effects of warming and drought could weaken, and even reverse, the positive effects of warming on the vegetation greening and productivity^33^. Therefore, the balance between temperature and moisture is an important interaction regulating spring plant growth.

The phenological response on the Qinghai-Tibetan Plateau may more closely resemble the responses to climate change in Mediterranean climatic systems than those in other cold regions. Substantial evidence has demonstrated that the spring phenology has advanced across the world, particularly in the cold regions, as a consequence of warming^34^,^35^,^36^,^37^. Mamolos *et al*.^38^ conducted an experiment to measure the phenology and productivity changes under drought conditions within the context of the strong seasonality of precipitation in the Mediterranean grasslands. Their results showed that the early-season grasses were more drought sensitive than other species. In other words, spring drought can restrict the growth of early-season grasses, delaying the onset of vegetation phenology. Similarly, Swarbreck *et al*.^39^ studied the impact of warming on the phenology and productivity of a Californian grassland system. They showed that in wet years, biomass production increased and phenology was earlier than in dry years. These results indicate that the effects of temperature and moisture are combined under warming conditions. In this sense, the grasslands of the Qinghai-Tibetan Plateau are responding to climatic changes more like Mediterranean grasslands than like other alpine systems.

The projection of future climate change scenarios forecasts that the climate on the central Qinghai-Tibetan Plateau will continue to become warmer and more arid^19^. Therefore, the spring phenology of the alpine grasslands on the central Qinghai-Tibetan Plateau could be further delayed in future years, which would change the allocation of water and nutrient resources, exacerbating the lack of forage for spring grazing. However, the net result of warming is a net increase in NEE, which could have complex effects on the grazing of both domestic and wild animals.

## Methods

### Regional Measurement

#### Climate data

The climate data for Nagqu County were obtained from the China Meteorological Data Sharing Service System of the China Meteorological Administration.

#### Green-up date calculation

We analyzed the region-wide changes in the green-up date on the central Qinghai-Tibetan Plateau (Nagqu County, characterized as an alpine meadow region) from 1982 to 2013 using the Normalized Difference Vegetation Index (NDVI) ratio method^23^,^40^. Our NDVI dataset was derived from imagery obtained from the Advanced Very High Resolution Radiometer (AVHRR) with a spatial and temporal resolutions of 8 km and 15 days, respectively. The NDVI ratio is calculated with the following equation: NDVI_ratio_ = (NDVI-NDVI_min_)/(NDVI_max_-NDVI_min_), where NDVI_ratio_ is the range from 0 to 1, NDVI is the NDVI value at a certain time, and NDVI_max_ and NDVI_min_ are the annual maximum and minimum NDVI values, respectively^40^. Based on our ground observational data collected on the central Qinghai-Tibetan Plateau and during other regional work^14^,^23^,^24^,^40^ on the Qinghai-Tibetan Plateau, we selected an NDVI threshold of 0.4 to estimate the green-up date on the central Qinghai-Tibetan Plateau.

### Field Experiment

#### Site and experimental design

We conducted a warming experiment in Nagqu County, Nagqu Prefecture, Tibet Autonomous Region, China (31.441°N, 92.017°E; 4500 m a.s.l.). The mean annual temperature is −1.2 °C, the annual precipitation is 431.7 mm, and more than 90% of the annual rainfall in the area occurs during the growing season (May–September), and is the main determinant of productivity^29^. Alpine meadow is the predominant plant community in the region. The dominant species are *Kobresia pygmaea, Carex moorcroftii*, and *Poa pratensis, Potentilla acaulis*, and *Oxytropis ochrocephala*. The experimental site was in a wet meadow in which the cover of *Kobresia pygmaea* is greater than 50%. The site was on a uniformly on a flat area. The soil bulk density was 1.01 g cm^−3^ with pH 7.05. The soil organic C, total C, total N, and total P contents of the soil were 41.39, 49.84, 6.78, and 1.43 g kg^−1^, respectively^41^.

Open top chambers (OTCs) were used to create sites of whole-year warming in our experiment. The OTCs were made of solar-radiation-transmitting materials and were cylindrical, with the height of 0.45 m, a diameter of 1.20 m at ground height, and a diameter of 0.65 m at the maximum height. Two types of OTCs were used in this study. One was the general type described above. The second modified type had a fan to reduce the heating effect. We began the whole-year warming experiment in July 2011 with four replicates of each of three treatment types: warming plots (W, use of general type of OTCs), limited warming plots (LW, modified OTCs), and control plots (CP), giving a total of 12 plots. Placing the chambers on the ground and staking them down resulted in minimal vegetation damage, and no analogous disturbance was imposed on the control plots. We report our results up to the end of 2014.

A 2500 m^2^ experimental area was fenced in 2010 and was not grazed or mown during the experimental period. All OTCs were approximately 3 m apart. Samples of plant growth were collected from a fixed area of 30 × 30 cm^2^ within the chambers or at the control locations. The chambers and control locations were randomized within a single area in this homogeneous environment.

#### Field measurements

Chamber-based measurements of NEE were made. We used the EM50 Data Collection System (Decagon Devices, Inc., NE, USA) for the microclimate measurements. The data were collected at 30 min intervals throughout the year. We used a portable photosynthesis system (Li-6400; LI-COR Inc., Lincoln, NE, USA) and the transparent static chamber method to measure the net ecosystem CO_2_ exchange (NEE) of each plot during the growing seasons from 2012 to 2014.

Before the experiment, we embedded each plot area with a plastic base (30 × 30 cm^2^) for measurement. We measured NEE three times per month, between 10:00 and 12:00 on sunny days from May through to September (the growing season). To make the measurements, we placed a transparent polyethylene chamber (30 cm × 30 cm × 40 cm) on the base of each plot, and placed a fan on the roof of each chamber, to mix the gases inside it, and measured NEE over a period of 90 seconds.

Environmental data were collected from fixed locations outside, but adjacent to, the plots used to measure NEE. Soil probes were used to measure the soil temperature and moisture. We measured the soil temperature and soil water content at a depth of 5 cm, where the main roots are distributed. Similarly, a fixed location approximately 0.2 m above the ground and 0.3 m for a chamber edge was used to measure air temperature measurements. We used soil temperature as our measure of the climate treatment effect size in the warmed versus control plots.

#### Data analysis

A series of random slope–mixed (soil moisture was dependent on the date of the measurement) effects models reflecting differing hypotheses regarding predictors of NEE were fitted to the data using the nlme package in Program R (v. 3.11, ref. 42). The effects modeled included the soil temperature, soil water content, change in soil water content, and the interaction between soil temperature and soil water content. Before the models were implemented, our predictors were standardized and rescaled to a mean of zero and unit variance^43^ in order to give equal potential weight to variables measured in different units (e.g., temperature and percentage change in the soil water content). Model selection based on Akaike’s information criterion (*AIC*) was used to identify the “best” models amongst the candidate set of nested *a priori* hypotheses^43^. Models with *AIC* difference (∆*AIC*) values greater than 4.0 were considered to best approximated the data and were used to calculate the model-averaged regression coefficients and unconditional standard errors^44^. The model-averaged regression coefficients were divided by their standard errors to calculate the *Z*-statistic for each parameter. Predictors with a *Z*-score >|2.0| were considered strong predictors of NEE^45^.

The differences in soil temperature and soil moisture between each plot were examined with repeated measures analysis of variance (RMANOVA), as were the seasonal variations of NEE. A Pearson correlation analysis was used to determine the correlation indices between the NEE and the soil environmental factors (soil temperature and soil moisture). We used a linear regression analysis to examine the relationships between the green-up date and climate attributes. These analyses were conducted with IBM SPSS Statistics version 20.0.

## Additional Information

**How to cite this article**: Ganjurjav, H. *et al*. Complex responses of spring vegetation growth to climate in a moisture-limited alpine meadow. *Sci. Rep.* **6**, 23356; doi: 10.1038/srep23356 (2016).

## Figures and Tables

**Table 1:** The *P* value of regressions of the mean air temperature (T), precipitation (P), and aridity index (AI) changes in each month from 1982 to 2013.

	Annal	Jan.	Feb.	Mar.	Apr.	May	Jun.	Jul.	Aug.	Sep.	Oct.	Nov.	Dec.
T	**<0.001**	**0.008**	**0.003**	**0.017**	**0.002**	0.155	0.151	**<0.001**	**<0.001**	**0.003**	0.340	**0.041**	**0.001**
P	0.451	0.391	−0.590	0.069	−0.992	**0.004**	−0.367	0.852	0.543	−0.140	**0.043**	−0.277	**−0.008**
AI	0.201	0.690	0.956	0.245	0.625	**0.011**	−0.298	−0.852	0.794	−0.082	0.077	−0.117	**0.019**

Positive values represent increasing trends, while negative values indicate decreasing trends.

**Table 2:** Correlation between the green-up date and twelve climatic attributes in central Qinghai-Tibetan Plateau from 1982 to 2013.

Factors		coeff	r^2^	p
**Temperature**	**MAT**	−0.032	<0.001	0.974
	**MWST**	−0.059	<0.001	0.942
	**Apr. T**	0.141	0.002	0.831
	**May T**	1.04	0.095	0.086
**Precipitation**	**MAP**	−0.002	0.001	0.869
	**MWSP**	**−0.06**	**0.15**	**0.027**
	**Apr. P**	−0.012	0.001	0.893
	**May P**	−0.062	0.118	0.054
**Aridity index**	**AAI**	−0.19	0.002	0.818
	**WSAI**	0.188	0.05	0.199
	**Apr. AI**	−0.05	<0.001	0.948
	**May AI**	**−0.90**	**0.14**	**0.036**

The pre-growing and early growing season temperature and precipitation attributes are presented. The climate attributes include: mean annual temperature (MAT), Mean winter/springtime temperature (Nov–May, MWST), Mean April temperature (Apr. T), Mean May temperature (May T), Mean annual precipitation (MAP), Mean winter/springtime precipitation (Nov–May, MWSP), Mean April precipitation (Apr. P), Mean May precipitation (May P), Annual aridity index (AAI), winter/springtime aridity index (WSAI), April aridity index (Apr. AI) and May aridity index (May AI).

**Table 3:** Model selection results for random-slope models of Net ecosystem CO_2_ exchange (NEE) for the central Qinghai-Tibetan Plateau.

Model	*K*^1^	AIC^2^	ΔAIC^3^	*w*^4^
*Early Growing Season*				
NEE = SWC + ST + ∆SWC	11	247.98	0.00	0.49
NEE = SWC + ∆SWC	10	248.95	0.97	0.30
NEE = SWC + ST + ∆SWC + ST:SWC	12	249.89	1.91	0.19
NEE = ∆SWC	9	254.97	6.99	0.01
NEE = ST	9	355.04	107.06	0.00
NEE = .	8	369.15	121.17	0.00
NEE = SWC	9	370.70	122.72	0.00
*Late Growing Season*				
NEE = SWC + ∆SWC + ST:SWC	12	967.39	0.00	0.66
NEE = SWC + ST + ∆SWC + ST:SWC	11	968.69	1.30	0.34
NEE = SWC + ST:SWC	10	1033.05	65.66	0.00
NEE = ST	9	1035.70	68.31	0.00
NEE = SWC	9	1061.69	94.30	0.00
NEE = .	8	1064.79	97.40	0.00

Modeled effects include soil temperature (ST), soil water content (SWC), change in soil water content (∆SWC, estimated as SWCt – SWCt-1), and the interaction of soil temperature and soil water content (ST:SWC) for both early- and late-growing seasons. All covariates were standardized and rescaled to a mean of 0 and unit variance. Results for the Null (.) model are presented to provide an indication of model performance relative to a model containing no covariates.

^1^Total number of model parameters, including those used to estimate the random effect of day on SWC.

^2^Akaike’s Information Criterion.

^3^AIC difference value.

^4^AIC model weight.

**Table 4:** Model-averaged regression coefficients 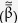, unconditional standard errors (SE) and Z-statistics (Z) for covariates used to predict Net ecosystem CO_2_ exchange (NEE) for the central Qinghai-Tibetan Plateau.

Predictor	Early Growing Season			Late Growing Season		
	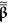	SE	*Z*^*1*^	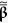	SE	*Z*^*1*^
Soil water content	0.74	0.27	2.74	0.97	0.47	2.07
Soil temperature	0.13	0.13	0.98	0.09	0.17	0.51
Change in soil water content	−0.43	0.18	−2.48	−0.19	0.26	−0.71
Interaction between soil water content and soil temperature	—	—	—	−0.51	0.33	−1.54
INTERCEPT	0.59	0.22	2.67	6.20	0.37	16.71

Parameter estimates are based on standardized and rescaled values for each covariate. Variables that were not estimated because they were absent from the best model set are denoted as “—“.
